# Case Report: Pulmonary reperfusion injury with bilateral bronchial cast thrombosis after VA-ECMO-bridged catheter-based reperfusion for cardiac arrest caused by high-risk pulmonary embolism

**DOI:** 10.3389/fcvm.2026.1970263

**Published:** 2026-10-05

**Authors:** Guanshuai Chen, Fanxiang Chen, Ruoyu Li

**Affiliations:** Department of Intensive Care Medicine, Dongguan Traditional Chinese Medicine Hospital, Dongguan, China

**Keywords:** bronchial cast thrombus, cardiac arrest, case report, cryoextraction, electrical impedance tomography, high-risk pulmonary embolism, veno-arterial extracorporeal membrane oxygenation (VA-ECMO)

## Abstract

High-risk pulmonary embolism (PE) can lead to acute right ventricular failure, obstructive shock, and cardiac arrest. Veno-arterial extracorporeal membrane oxygenation (VA-ECMO) provides bridging life support, buying time for imaging confirmation and reperfusion. However, reperfusion injury, pulmonary hemorrhage, and airway thrombotic obstruction may follow pulmonary artery reperfusion. Severe airway obstruction caused by bronchial cast thrombi is rare and challenging. We report a woman in her 60s awaiting surgery for a left femoral neck fracture who developed preoperative cardiac arrest due to high-risk PE. After VA-ECMO bridging and staged catheter-based pulmonary reperfusion, the patient developed reperfusion injury and bilateral bronchial cast thrombosis, managed by repeated bronchoscopy with cryoextraction. Electrical impedance tomography (EIT) monitored ventilation and perfusion. The patient was weaned from VA-ECMO and invasive ventilation but remained in a persistent vegetative state due to hypoxic-ischemic encephalopathy. This case suggests that in cardiac arrest caused by high-risk PE, VA-ECMO can buy time for diagnosis and reperfusion; however, reperfusion injury after recanalization can cause severe hemorrhage, with blood coagulating within the airways into bronchial cast thrombi and life-threatening obstruction. Bronchoscopy with cryoextraction effectively clears tenacious airway cast thrombi. EIT assists ventilation/perfusion monitoring during complex VA-ECMO support. Even when cardiopulmonary function recovers, hypoxic-ischemic brain injury may determine long-term prognosis.

## 1 Introduction

Hip fracture patients have a significantly increased risk of venous thromboembolism (VTE), and high-risk pulmonary embolism (PE) can rapidly progress to acute right ventricular failure, obstructive shock, and cardiac arrest (1, 2). In circulatory collapse or arrest, anticoagulation alone cannot promptly reverse hemodynamic failure. VA-ECMO provides mechanical circulatory and oxygenation support, maintains vital organ perfusion, and buys time for imaging and reperfusion (3, 4). Recently, structured algorithms have been proposed for high-risk massive PE requiring mechanical support (5).

Catheter-based intervention is an important reperfusion strategy for selected high-risk PE; however, recanalization of occluded pulmonary arteries may cause reperfusion injury, in severe cases alveolar hemorrhage and worsening oxygenation (6, 7). Blood entering and coagulating within the airways may form tenacious bronchial cast thrombi, causing severe obstruction that conventional suction often fails to clear; cryoextraction—adhering the thrombus to a cryoprobe for *en bloc* removal—offers advantages (8, 9).

Electrical impedance tomography (EIT) is a bedside, real-time, non-invasive technique for monitoring pulmonary ventilation and perfusion (10, 11), facilitating dynamic assessment in complex settings such as ECMO, hemorrhage, and airway obstruction.

We report a woman in her 60s awaiting left femoral neck fracture surgery who developed preoperative cardiac arrest due to high-risk PE. Following VA-ECMO bridging and staged catheter-based reperfusion, the patient developed pulmonary reperfusion injury with bilateral bronchial cast thrombosis, cleared by repeated bronchoscopy with cryoextraction; however, hypoxic-ischemic encephalopathy ensued, leaving a persistent vegetative state.

## 2 Case presentation

### 2.1 General information and admission

A woman in her 60s presented with left hip pain and limited mobility after a left-sided fall while walking 5 h prior, without loss of consciousness, chest pain, or dyspnea at injury. Computed tomography (CT) at a local hospital revealed a left femoral neck fracture, and the patient was transferred to our institution.

On admission, the patient was conscious and oriented, with an acute painful appearance. Vital signs: temperature 36.3 °C, heart rate 83/min, respiratory rate 20/min, blood pressure 158/88 mmHg, and oxygen saturation (SO₂) 97% on nasal cannula. Cardiopulmonary and abdominal examination was unremarkable. The left hip showed mild swelling, maximal inguinal midpoint tenderness, palpable bony crepitus, loss of weight-bearing, and a positive heel-tap test. Distal pulses and toe movement were intact.

The patient had a 6-year history of hypertension on telmisartan, reportedly well-controlled. There was no diabetes mellitus, coronary artery disease, cerebrovascular disease, prior surgery, or thromboembolic events. She was a never-smoker and did not consume alcohol; she was retired and lived with her family. There was no family history of thromboembolic or cardiovascular disease, and screening for inherited thrombophilia was negative. Allergies: penicillin and sulfonamides. The Caprini score was 10, indicating extremely high VTE risk (12). Enoxaparin sodium was given preoperatively for thromboprophylaxis. The chronological clinical course is summarized in Table 1.

**Table 1:** Chronological clinical course of the patient.

Timepoint	Clinical event	Examination/findings	Intervention	Outcome
Admission day 1	Left hip pain after fall	CT: left femoral neck fracture; Caprini score 10	Analgesia, immobilization, preoperative assessment; enoxaparin prophylaxis	Awaiting surgery
Admission day 6	Preoperative cardiac arrest	ECG: complete RBBB; echocardiography: suspected PE; lower-extremity ultrasound: left peroneal, posterior tibial, and calf intermuscular vein thrombosis	CPR, epinephrine 14 mg in total, defibrillation 200 J; VA-ECMO establishment (37 min from arrest to full ECMO flow)	Return of spontaneous circulation; VA-ECMO support established
Admission day 7	High-risk PE confirmed; first thrombectomy	CTA: bilateral main and branch pulmonary artery embolism; DSA: bilateral filling defects (Figures 1A,B)	Percutaneous pulmonary thrombectomy; urokinase 200,000 U local thrombolysis	Partial flow restoration (Figures 1C,D)
Admission day 8	ECMO and mechanical ventilation support	D-dimer elevated; PaO₂/FiO₂ ∼100 mmHg; Hb 61 g/L	Anticoagulation (heparin), respiratory and circulatory support	Stabilized on ECMO
Admission day 10	Interval reassessment	CTPA: persistent multiple filling defects in bilateral pulmonary artery trunks and lobar/segmental branches; persistent left pulmonary artery occlusion	Vascular intervention consultation; decision for staged repeat thrombectomy	Second intervention scheduled
Admission day 11	Second left pulmonary artery thrombectomy	Angiography: left pulmonary artery and lower-lobe occlusion	Thrombolysis, thrombectomy, balloon angioplasty	Partial left pulmonary artery flow restoration
Admission day 11 (post-procedure)	Pulmonary reperfusion injury; bilateral bronchial cast thrombosis	Bronchoscopy: tracheal tube obstruction extending to bilateral main bronchi (Figures 2A–C); ABG: pH 7.150, PaCO₂ 65, PaO₂ 52, SO₂ 74%	Tracheal tube change; bronchoscopic forceps extraction, suction, lavage, local hemostasis	Initial airway reopening; oxygenation improved (ABG: pH 7.340, PaCO₂ 32, PaO₂ 100, SO₂ 95%)
Admission day 15	Bronchoscopic cryoextraction (second session)	Left bronchus 95% thrombus obstruction; cryoextraction performed (Figure 2E)	Bronchoscopic cryoextraction; epinephrine spray; iced saline lavage	Left bronchus partially patent; remaining thrombus deferred
Admission day 16	Anticoagulation de-escalation	Airway bleeding bronchoscopically controlled	Transition from intravenous heparin to subcutaneous enoxaparin 2,000 U daily	No recurrent bleeding
Admission day 17	Bronchoscopic cryoextraction (third session)	Left upper-lobe orifice thrombus cleared (Figures 2F,G)	Bronchoscopic cryoextraction (third session)	Left upper- and lower-lobe bronchi patent; no active bleeding
Admission day 18	Hemodynamic stabilization	Stable hemodynamics; lactate normalized	VA-ECMO weaning and decannulation	Successful VA-ECMO decannulation
Admission day 19	EIT monitoring (first day off ECMO)	EIT: VQMatch improved from 52.01% to 68.18% (Figures 3D–F)	—	Improved ventilation/perfusion matching
Admission day 28	Long-term airway management	—	Tracheostomy	—
Admission day 29	Respiratory improvement	—	Mechanical ventilation weaning	—
Admission day 38	Neuroimaging follow-up	MRI: cerebral atrophy, Fazekas grade 1 white matter changes, no acute infarcts; MRA: cerebral arteriosclerosis	—	Persistent vegetative state
Admission day 55 onward	Neurological rehabilitation	EEG: abnormal; hyperbaric oxygen therapy initiated	Hyperbaric oxygen therapy	—
Admission day 75	Neurological assessment	EEG: slow background, poor regulation and amplitude	Supportive care	Persistent vegetative state (GCS E4V1M1, CPC 4)

ABG, arterial blood gas; CPR, cardiopulmonary resuscitation; CT, computed tomography; CTA, computed tomography angiography; CTPA, computed tomography pulmonary angiography; DSA, digital subtraction angiography; DVT, deep vein thrombosis; ECG, electrocardiography; EEG, electroencephalography; EIT, electrical impedance tomography; Hb, hemoglobin; PaO₂/FiO₂, ratio of arterial partial pressure of oxygen to fraction of inspired oxygen; PE, pulmonary embolism; RBBB, right bundle branch block; SC, subcutaneous; SO₂, oxygen saturation; VA-ECMO, veno-arterial extracorporeal membrane oxygenation.

### 2.2 Preoperative sudden cardiac and respiratory arrest and VA-ECMO support

On hospital day 6, on the operating table during surgical preparation, sudden respiratory and cardiac arrest occurred. At 12:50, profound bradycardia (30–40/min) with unmeasurable blood pressure indicated ineffective circulation; 1 mg epinephrine was given intravenously with immediate chest compressions and endotracheal intubation. At 12:52, ventricular fibrillation was identified; immediate 200-J biphasic defibrillation was delivered and a second 1-mg bolus was given, followed by twelve further 1-mg boluses at approximately 3-minute intervals (cumulative 14 mg; Supplementary Table S2). Full VA-ECMO flow was established at 13:29, 37 min after confirmed arrest (39 min after the onset of ineffective circulation), and spontaneous sinus rhythm was restored at 13:33. The no-flow interval was essentially zero (compressions begun immediately upon recognition of ineffective circulation); the low-flow interval, from the onset of ineffective circulation to full VA-ECMO flow, was 39 min. Bedside electrocardiography showed complete right bundle branch block, and echocardiography revealed an abnormal echogenic mass at the left pulmonary artery ostium, suggesting acute PE. Bilateral lower-extremity venous ultrasonography revealed thrombi in the left peroneal and posterior tibial veins and calf intermuscular veins.

### 2.3 Confirmation of pulmonary embolism and interventional therapy

Vital signs gradually stabilized on VA-ECMO. On day 7, computed tomography angiography (CTA) confirmed embolism of the bilateral main pulmonary arteries and multiple branches. Same-day emergent pulmonary angiography via the femoral vein showed bilateral filling defects (Figures 1A, B); after local catheter-directed thrombolysis (200,000 U urokinase) and thrombus aspiration in the bilateral main and lower-lobe arteries, several red thrombi were removed, with partial flow restoration on repeat angiography (Figures 1C, D).

**Figure 1:**
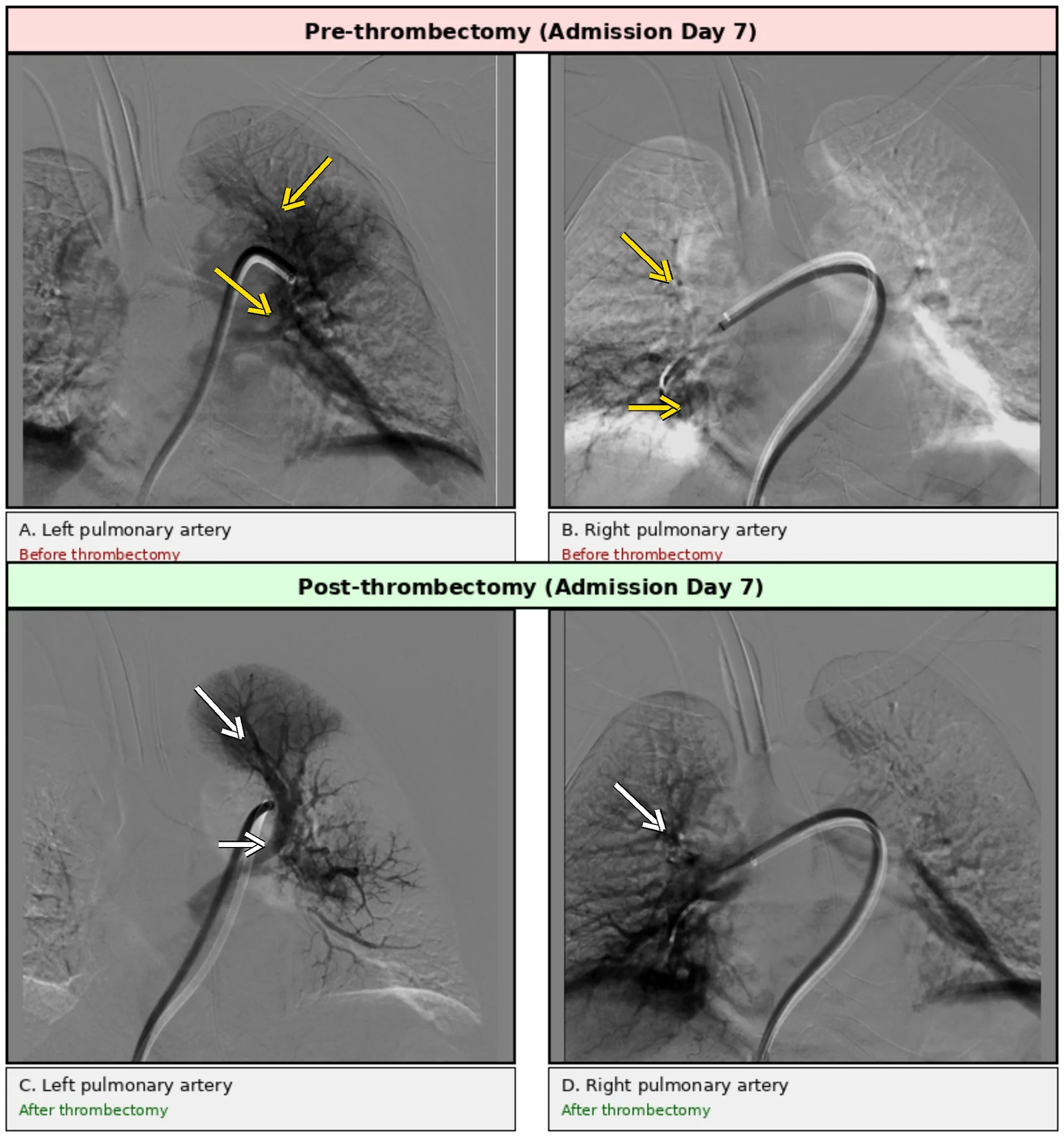
Pulmonary angiography. **(A,B)** Bilateral pulmonary artery filling defects before intervention (hospital day 7); yellow arrows indicate filling defects with absent or markedly reduced distal flow. **(C,D)** Reduced extent of filling defects with partially restored branch flow after catheter-directed thrombolysis and aspiration thrombectomy; white arrows indicate recanalized branches with restored antegrade flow.

After the first intervention, hemodynamics remained stable with partially restored branch flow; given severe anemia (hemoglobin 61 g/L on day 8) and the need to balance anticoagulation against bleeding, a staged reperfusion strategy was adopted. Repeat CT pulmonary angiography on day 10 showed persistent multiple filling defects in the bilateral pulmonary artery trunks and lobar/segmental branches with persistent left pulmonary artery occlusion; multidisciplinary consultation recommended repeat intervention.

On day 11, a second left pulmonary artery reperfusion was performed under VA-ECMO for significant residual thrombus with impaired perfusion. Angiography showed multiple thrombi in the left main and lower-lobe pulmonary arteries with complete occlusion. A 6 mm × 40 mm balloon was first dilated in the left lower-lobe artery, followed by mechanical aspiration thrombectomy (10-F and 8-F aspiration catheter system with microcatheter) and local thrombolysis (200,000 U urokinase). Post-dilation angiography showed partial flow restoration in the left pulmonary artery trunk and lower-lobe branches, without extravasation, dissection, or abrupt cut-off; an indwelling thrombolysis catheter was left for continued urokinase infusion after return to the intensive care unit (ICU).

### 2.4 Pulmonary reperfusion injury and bronchial cast thrombosis

After the second intervention on day 11 (14:20–16:20, with balloon dilation as the first step; return to the ICU at approximately 17:00), bloody secretions were first noted from the endotracheal tube at 19:10—2 h 50 min after completion of the procedure—with deteriorating ventilatory parameters, suggesting pulmonary reperfusion injury and hemorrhage. Arterial blood gas (ABG): pH 7.150, PaCO₂ 65 mmHg, PaO₂ 52 mmHg, SO₂ 74%.

Bedside flexible bronchoscopy revealed massive bloody secretions and thrombi obstructing the endotracheal tube, trachea, carina, and bilateral main bronchi, causing severe airway obstruction (Figures 2A–C). After tube replacement, forceps extraction, suction, bronchoalveolar lavage, and local hemostasis gradually reopened the central and lobar bronchi, with markedly improved oxygenation (Figures 2A–D).

**Figure 2:**
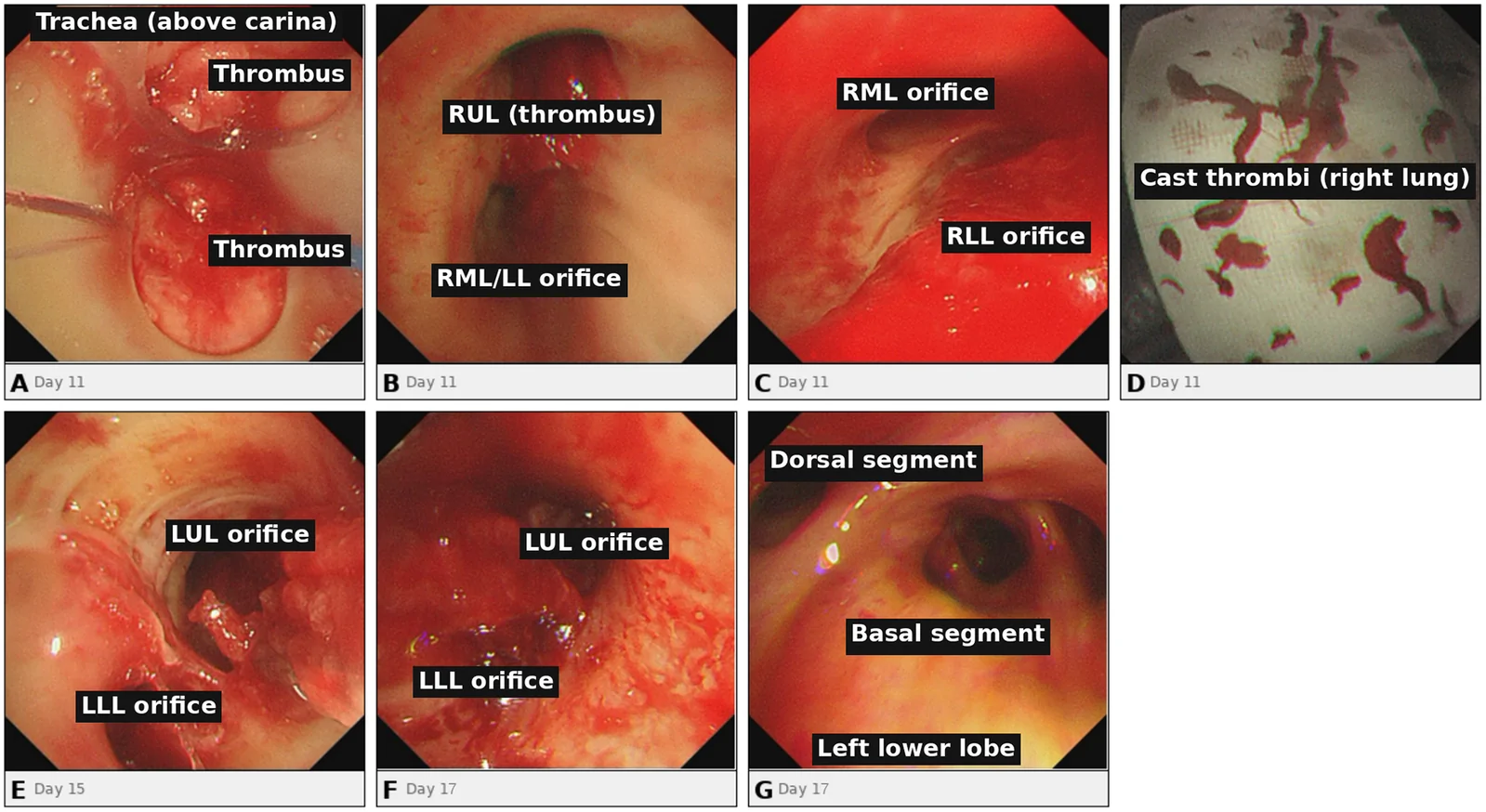
Bronchoscopic findings and thrombus removal. **(A)** Supracarinal trachea with thrombi (hospital day 11). **(B)** Right upper-lobe (RUL) bronchial orifice occluded by thrombus; the right middle-/lower-lobe (RML/LL) orifice is seen below (hospital day 11). **(C)** RML and RLL orifices with massive bloody secretions (hospital day 11). **(D)** Bronchial cast thrombi extracted from the right lung (hospital day 11). **(E)** Left upper-lobe (LUL) and lower-lobe (LLL) orifices with residual thrombus during cryoextraction (hospital day 15). **(F)** LUL and LLL orifices during the third thrombus-removal session (hospital day 17). **(G)** Patent dorsal and basal segments of the left lower lobe after clearance (hospital day 17). RUL, right upper lobe; RML, right middle lobe; RLL, right lower lobe; LUL, left upper lobe; LLL, left lower lobe.

On day 15, bronchoscopy with cryoextraction was repeated (Figure 2E): thrombi obstructing approximately 95% of the left bronchial orifice were frozen and removed piecemeal, with epinephrine spray and iced saline for mucosal oozing. As further removal carried high risk of recurrent airway bleeding and ventilation had improved markedly, the remaining thrombi were left for elective management.

On day 17, a further session (Figures 2F,G): thrombi obstructing the left upper-lobe orifice were cryoextracted in several passes, gradually exposing the left upper- and lower-lobe orifices; a small dark-red thrombus in the dorsal segment of the left lower lobe was suctioned clear, and the basal segments were patent without active bleeding.

### 2.5 Bedside EIT monitoring of ventilation and perfusion

Dynamic bedside EIT monitoring was performed during VA-ECMO and invasive mechanical ventilation; the complete series is provided in Supplementary Table S3. Because saline bolus-based perfusion maps under VA-ECMO reflect residual native transpulmonary flow rather than total pulmonary perfusion, on-ECMO indices were interpreted qualitatively (regional distribution and within-patient trends) (13, 14). On day 7 (ECMO day 2) (Figures 3A–C), EIT showed Shunt% 4.95%, DeadSpace% 43.03%, and VQMatch% 52.01%. After the first and second bronchoscopic clearance sessions (days 11 and 15) and while still on full VA-ECMO, VQMatch% had improved to 59.45–72.73% (days 13–16). On day 19 (first day off ECMO, after the third clearance session on day 17) (Figures 3D–F), measurements showed Shunt% 5.3%, DeadSpace% 26.52%, and VQMatch% 68.18%.

**Figure 3:**
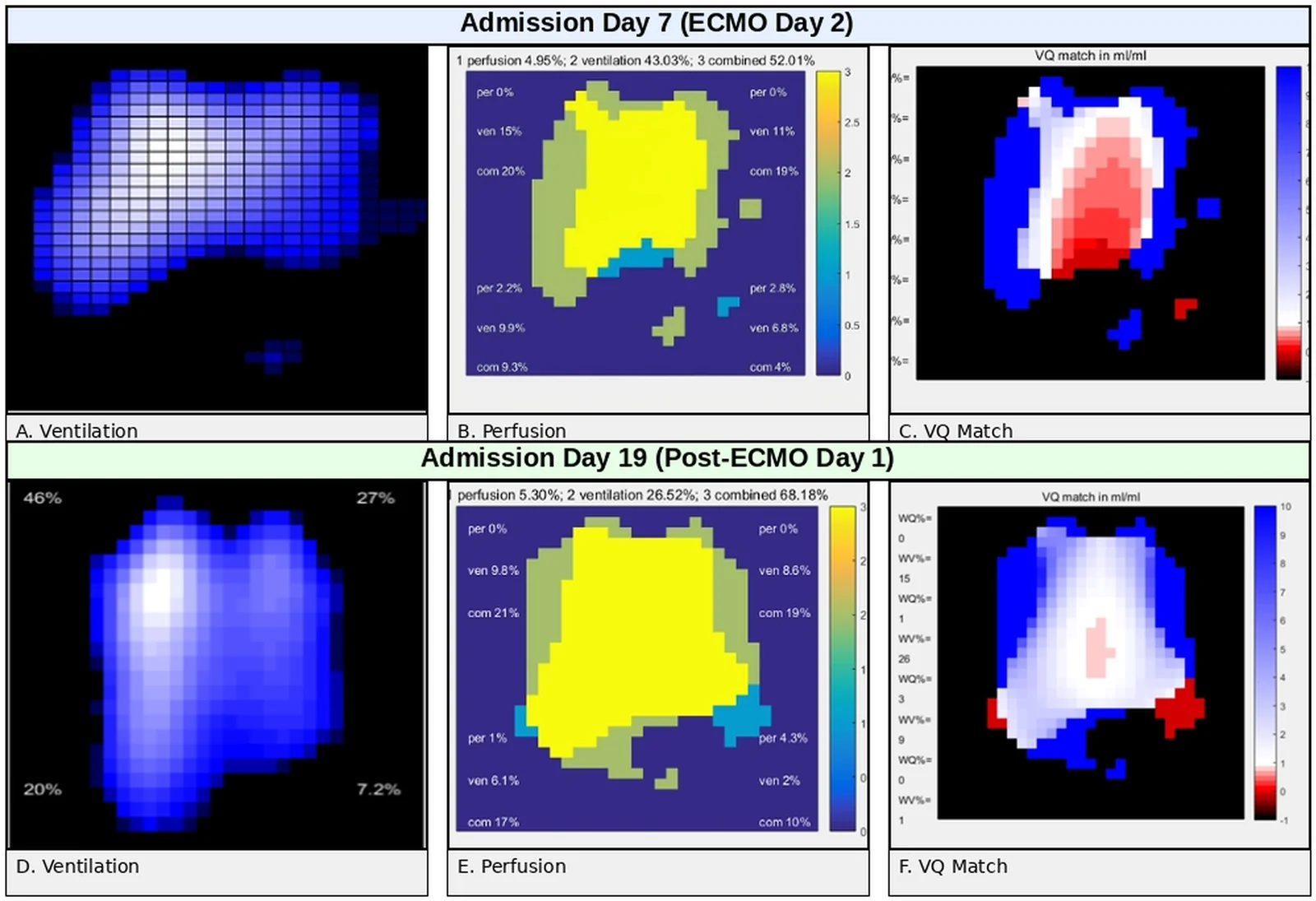
Bedside electrical impedance tomography (EIT) monitoring. **(A–C)** Hospital day 7 (ECMO day 2): Shunt 4.95%, DeadSpace 43.03%, VQMatch 52.01%. **(D–F)** Hospital day 19 (first day after ECMO discontinuation): Shunt 5.3%, DeadSpace 26.52%, VQMatch 68.18%.

### 2.6 Anticoagulation, ECMO management, and outcome

Continuous intravenous unfractionated heparin was given from day 6 to 15, adjusted by bedside activated clotting time (ACT) and serial coagulation testing (Supplementary Table S1). The periprocedural heparin targets during combined VA-ECMO and catheter-directed reperfusion were activated partial thromboplastin time (APTT) 50–70 s and bedside ACT 140–160 s—a bleeding-risk-adapted regimen per institutional protocol and Extracorporeal Life Support Organization (ELSO) guidance (15)—with further deliberate reduction during active bleeding. On day 11, the APTT at the time of hemorrhage (74.6 s) was at the upper boundary of the target range, marginally above the 70-s upper limit but not meaningfully supratherapeutic. ACT ranged 104–181 s during the ECMO run. Serial APTT was 94.4–99.7 s initially (ECMO days 1–2) and was subsequently titrated toward the target range. During re-titration after the post-hemorrhage dose reduction, APTT transiently overshot to 108 s on day 14; this was recognized the same day, the heparin infusion was reduced, and APTT returned to 54.5 s the following day without recurrent bleeding (Supplementary Table S1). Anticoagulation remained essential given concurrent risks of PE progression, deep vein thrombosis, ECMO-circuit thrombosis, and residual thrombus. Heparin was promptly reduced after the hemorrhage (APTT 39.6 s on day 12) and titrated thereafter; protamine was not administered. On day 16, with airway bleeding bronchoscopically controlled and ECMO weaning approaching, anticoagulation was transitioned to subcutaneous enoxaparin 2,000 U daily as individualized de-escalation, consistent with ELSO recommendations for patients at high bleeding risk (15). The patient was weaned from VA-ECMO on day 18 after approximately 12 days.

### 2.7 Neurological outcome and follow-up

The patient developed hypoxic-ischemic encephalopathy after the arrest. Repeated bedside electroencephalography (EEG) showed poor wave amplitude. On day 75, formal EEG showed slow background activity with impaired regulation and amplitude modulation. Hyperbaric oxygen therapy began on day 55, but consciousness recovery remained unsatisfactory; the patient remained in a persistent vegetative state.

On day 38, brain magnetic resonance imaging (MRI), including diffusion-weighted imaging (DWI) and magnetic resonance angiography (MRA), revealed cerebral atrophy with widened sulci and mildly dilated ventricles, bilateral frontoparietal non-specific white matter changes (Fazekas grade 1) without restricted diffusion, and no acute infarcts. MRA showed cerebral arteriosclerosis with irregular walls and mild right internal carotid cavernous narrowing. Right mastoiditis was also noted. Latest Glasgow Coma Scale (GCS) score was E4 V1 M1 (GCS 6); Cerebral Performance Category (CPC) was 4. Follow-up was 6 months at submission. No further unexpected adverse events related to the interventions occurred during the remainder of hospitalization. The family requested continued hospitalization and conservative care.

## 3 Discussion

### 3.1 The bridging role of VA-ECMO in cardiac arrest caused by high-risk pulmonary embolism

When high-risk PE causes cardiac arrest, sudden pulmonary artery obstruction increases right ventricular afterload, leading to obstructive shock and circulatory collapse (2); conventional CPR is unlikely to restore effective circulation when pulmonary artery flow is severely obstructed (16). VA-ECMO provides circulatory and oxygenation support, unloads the right ventricle, and buys time for diagnosis and reperfusion (3, 4, 17).

### 3.2 A structured management algorithm for high-risk massive PE requiring mechanical circulatory support

Contemporary algorithms for high-risk massive PE recommend a stepwise approach with VA-ECMO as the central bridging platform (5). For cardiac arrest or refractory obstructive shock, immediate VA-ECMO is recommended and improves survival (5, 16). After stabilization, definitive imaging (CTA) and reperfusion follow: catheter-directed techniques are first-line, whereas surgical embolectomy is reserved for failed catheter therapy, contraindications to thrombolysis, or concomitant surgical indications (2, 5). When complete single-session recanalization is unsafe, staged intervention under continued ECMO is acceptable (5).

Our management was consistent with this framework: VA-ECMO was established at the bedside within 37 min of cardiac arrest—well within the early-initiation window (5)—allowing hemodynamic stabilization, day-7 CTA confirmation, catheter-directed thrombolysis with aspiration thrombectomy, and a staged second intervention on day 11 for persistent left pulmonary artery occlusion. The 4-day interval reflected deliberate staging—anemia correction, anticoagulation balancing, and repeat-CTA reassessment—rather than deferral; surgical embolectomy was not pursued given feasible catheter recanalization and the risks of sternotomy and bypass in the peri-fracture, bleeding-prone setting (5, 18).

### 3.3 Bronchial cast thrombosis and the technical value of cryoextraction

Sudden recanalization after prolonged pulmonary artery occlusion can cause reperfusion injury through endothelial injury, inflammation, capillary hyperpermeability, oxidative stress, and alveolar–capillary barrier disruption (6), manifesting as pulmonary edema, alveolar hemorrhage, and worsening oxygenation (7). Blood entering and coagulating within the airways may form dendritic cast thrombi along the bronchial tree that resist conventional suction (8, 9, 19).

In our patient, severe pulmonary hemorrhage followed partial flow restoration of the occluded left pulmonary artery on day 11, with blood coagulating within the trachea and bronchi into bilateral cast thrombi; cryoextraction proved effective for these large, tenacious casts that were difficult to grasp or suction (8, 9, 20).

The use of balloon dilation in this acute setting warrants discussion. Balloon pulmonary angioplasty is established for chronic thromboembolic pulmonary hypertension (7, 19); in acute PE it differs fundamentally, carrying risks of dissection, distal perforation, and severe alveolar hemorrhage. In our patient, the left lower-lobe artery was dilated with a 6 mm × 40 mm balloon—a conservative size for a lobar branch—only after the vessel remained completely occluded despite local thrombolysis; balloon-assisted thrombus maceration and dilation are recognized catheter-directed techniques for acute high-risk PE (2). The bilateral hemorrhage distribution, absent angiographic vascular injury, and delayed onset favor ischemia–reperfusion injury over focal perforation, although a mechanical component cannot be entirely excluded. Endobronchial bleeding was first noted 2 h 50 min after completion of the intervention and approximately 4 h after balloon dilation, with no bleeding during or immediately after the procedure; such delayed onset favors ischemia–reperfusion injury over mechanical perforation, which would be expected to bleed during or immediately after inflation (6, 7). Conservative balloon sizing, anticipation of reperfusion hemorrhage, and routine post-dilation angiography remain essential.

### 3.4 The monitoring value of EIT in complex critical pulmonary disease

EIT enables bedside, continuous, radiation-free monitoring of regional ventilation, including inhomogeneity, overdistension, collapse, and positive end-expiratory pressure (PEEP) responsiveness (10). Combined with hypertonic saline bolus injection, EIT can also assess lung perfusion and ventilation/perfusion matching (11). In our patient—with high-risk PE, reperfusion injury, pulmonary hemorrhage, airway obstruction, VA-ECMO, and invasive ventilation—EIT helped track ventilation improvement around airway clearance and perfusion changes after recanalization, informing ventilator adjustment and bronchoscopic intervention evaluation (21, 22).

### 3.5 Resuscitation burden, epinephrine exposure, and neurological outcome in extracorporeal cardiopulmonary resuscitation (E-CPR)

The unfavorable neurological outcome warrants candid discussion. The event was immediately recognized in-hospital: at 12:50, on the operating table during surgical preparation, profound bradycardia (30–40/min) with unmeasurable blood pressure prompted immediate CPR with endotracheal intubation, so the no-flow interval was essentially zero; nevertheless, the low-flow period—from the onset of ineffective circulation (12:50) to full VA-ECMO flow (13:29)—lasted 39 min. Cumulative epinephrine exposure was 14 mg: one bolus during the peri-arrest phase (12:50), a second immediately after defibrillation for ventricular fibrillation (12:52), and twelve subsequent boluses at approximately 3-minute intervals (12:55–13:28) until VA-ECMO flow was established (Supplementary Table S2). In high-risk PE, obstructive physiology limits the forward pulmonary flow conventional CPR can generate (16), so even guideline-concordant compressions may inadequately perfuse the brain.

Peri-arrest boluses were administered without delay because the clinical picture was indistinguishable from a pulseless, low-flow state, and the regimen was consistent with advanced life support. Although epinephrine remains a resuscitation cornerstone, high cumulative doses induce cerebral microvascular vasoconstriction; in the PARAMEDIC2 randomized trial, epinephrine improved 30-day survival but increased severe neurological impairment among survivors (23). This cumulative exposure plausibly contributed to the hypoxic-ischemic brain injury despite timely E-CPR. We acknowledge that peri-arrest bolus use is debated and that earlier ECMO activation might have limited exposure.

Neuroprognostication after cardiac arrest, particularly in E-CPR recipients, should be deferred and multimodal, integrating clinical examination, electrophysiology, neuroimaging, and biomarkers (24); in ECMO recipients, sedation and circuit factors may confound assessment. As described in Section 2.7, serial EEG and clinical assessment confirmed severe, persistent hypoxic-ischemic brain injury despite rehabilitation.

This case underscores that even technically successful E-CPR cannot reverse neurological injury sustained during low flow (25); minimizing no-flow/low-flow time, judicious epinephrine use, earlier ECMO activation, and early multimodal neurological assessment should be integral to E-CPR management.

### 3.6 Strengths and limitations

Strengths include rapid VA-ECMO establishment within 37 min of confirmed arrest, staged catheter-based reperfusion under ECMO, successful cryoextraction clearance of extensive bilateral bronchial cast thrombi, and dynamic bedside EIT monitoring. Several limitations exist. First, this is a single case with limited generalizability. Second, EIT perfusion findings during VA-ECMO require cautious interpretation. Saline bolus-based EIT perfusion imaging is a first-pass indicator-dilution technique; during peripheral VA-ECMO, part of the indicator is drained into the extracorporeal circuit before traversing the pulmonary circulation, so the maps reflect only residual native transpulmonary flow (13), and dead-space fraction and V/Q matching remain semi-quantitative, varying with ECMO blood flow independently of true perfusion changes (14). Serial EIT measurements (Supplementary Table S3) showed that most of the improvement in VQMatch% occurred during VA-ECMO after bronchoscopic clearance (52.01% on day 7 versus 59.45–72.73% on days 13–16, i.e., after the first and second clearance sessions on days 11 and 15, before weaning on day 18), and that the first off-ECMO value (68.18% on day 19, after the third clearance session on day 17) lay within the on-ECMO post-clearance range. The improvement therefore predominantly reflects bronchial clearance and recanalization rather than disappearance of the ECMO-related steal artifact at weaning. Nevertheless, absolute on-ECMO perfusion values remain semi-quantitative (13, 14), and single time points should be interpreted in the context of within-patient trends. Although the diffuse bilateral hemorrhage distribution, absent angiographic vascular injury, and delayed onset favor reperfusion injury, a mechanical component from balloon dilation cannot be entirely excluded. Third, the optimal anticoagulation strategy balancing reperfusion, thrombosis, and bleeding remains uncertain; our individualized de-escalation was consistent with ELSO guidance (15), but monitoring relied on ACT and APTT rather than Anti-Xa assays, and ideal monitoring and targets during combined VA-ECMO and catheter-directed thrombolysis require further study.

## 4 Patient perspective

The patient remains in a persistent vegetative state and cannot provide a personal perspective. The family/legal representative expressed gratitude for the multidisciplinary team's life-saving efforts and agreed to publication, hoping to inform the management of similar patients.

## 5 Conclusion

For cardiac arrest caused by high-risk PE, VA-ECMO can bridge to diagnosis and interventional reperfusion (3). Pulmonary reperfusion injury with secondary bronchial cast thrombosis should be anticipated after recanalization; bronchoscopy with cryoextraction is an effective rescue technique for tenacious airway casts (8). EIT is a useful bedside monitoring adjunct in complex cardiopulmonary support. Even when cardiopulmonary function recovers, hypoxic-ischemic brain injury may determine long-term prognosis (25), and the efficacy of rehabilitative treatments such as hyperbaric oxygen requires further high-quality evidence (26).

## Data Availability

The original contributions presented in the study are included in the article/Supplementary Material, further inquiries can be directed to the corresponding author.
