# Supplementary material for: Case Report: Pulmonary reperfusion injury with bilateral bronchial cast thrombosis after VA-ECMO-bridged catheter-based reperfusion for cardiac arrest caused by high-risk pulmonary embolism
Source: Front Cardiovasc Med. 2026 Oct 5;13:1970263. doi: 10.3389/fcvm.2026.1970263 (PMC13677917; doi:10.3389/fcvm.2026.1970263)
Supplement: Supplementary file 1 [file Table1.docx]

**Supplementary Material**

*Pulmonary reperfusion injury with bilateral bronchial cast thrombosis after VA-ECMO-bridged catheter-based reperfusion for cardiac arrest caused by high-risk pulmonary embolism*

Guanshuai Chen, Fanxiang Chen, Ruoyu Li (corresponding author) — Frontiers in Cardiovascular Medicine

**Supplementary Table S1.** Serial coagulation parameters during VA-ECMO support.

| **Hospital day** | **ECMO day** | **PT (s)** | **APTT (s)** | **INR** | **Fibrinogen (g/L)** | **D-dimer (mg/L)** | **Platelets (×10⁹/L)** | **ACT (s)** |
| --- | --- | --- | --- | --- | --- | --- | --- | --- |
| 6 | 1 | 19.3 | 94.43 | 1.81 | 2.41 | 4.06 | 128 | 152–169 |
| 7 | 2 | 16.5 | 99.73 | 1.46 | 2.97 | 12.66 | 91.5 | — |
| 8 | 3 | 15.3 | 48.87 | 1.32 | 3.3 | 8.84 | 72 | 104–119 |
| 9 | 4 | 14.57 | 69.07 | 1.24 | 4.69 | 4.65 | 63 | 161–181 |
| 10 | 5 | 14.8 | 63.65 | 1.27 | 4.61 | 8.05 | 74 | 145 |
| 11 | 6 | 16.33 | 74.57 | 1.45 | 3.27 | — | 60 | 171 |
| 12 | 7 | 15.7 | 39.55 | 1.38 | 3.08 | — | 38 | 174 |
| 13 | 8 | 14.95 | 48.75 | 1.28 | 2.92 | — | 58 | — |
| 14 | 9 | 16.93 | 108 | 1.52 | 2.66 | — | 70 | 153 |
| 15 | 10 | 16.5 | 54.5 | 1.47 | 3.25 | 4.78 | 82 | — |
| 16 | 11 | 15.7 | 35.6 | 1.37 | 3.93 | — | 77 | — |
| 17 | 12 | 16.2 | 39.4 | 1.43 | 4.62 | — | 86 | — |

APTT, activated partial thromboplastin time; INR, international normalized ratio; ACT, activated clotting time. ACT values are shown as the daily range when multiple measurements were available. Dashes indicate that the parameter was not measured on that day. Shaded rows mark hospital day 11 (onset of pulmonary hemorrhage) and day 12 (after heparin dose reduction). Heparin targets: APTT 50–70 s and ACT 140–160 s during combined VA-ECMO and catheter-directed reperfusion (bleeding-risk-adapted); targets were lowered further during active bleeding and after the day-11 hemorrhage. The day-14 APTT excursion (108 s) occurred during re-titration after deliberate sub-target anticoagulation for the day-11 hemorrhage; it was recognized the same day, the heparin dose was reduced, and APTT returned to target the following day without recurrent bleeding.
