# Supplementary material for: Case Report: Pulmonary reperfusion injury with bilateral bronchial cast thrombosis after VA-ECMO-bridged catheter-based reperfusion for cardiac arrest caused by high-risk pulmonary embolism
Source: Front Cardiovasc Med. 2026 Oct 5;13:1970263. doi: 10.3389/fcvm.2026.1970263 (PMC13677917; doi:10.3389/fcvm.2026.1970263)
Supplement: Supplementary file 2 [file Table2.docx]

**Supplementary Material**

*Pulmonary reperfusion injury with bilateral bronchial cast thrombosis after VA-ECMO-bridged catheter-based reperfusion for cardiac arrest caused by high-risk pulmonary embolism*

Guanshuai Chen, Fanxiang Chen, Ruoyu Li (corresponding author) — Frontiers in Cardiovascular Medicine

**Supplementary Table S2.** Resuscitation timeline with epinephrine bolus administration (hospital day 6).

| **Clock time** | **From onset (12:50)** | **Event** | **Bolus (IV)** | **Cumulative** |
| --- | --- | --- | --- | --- |
| 12:50 | 0 min | On the operating table under continuous monitoring: sudden unresponsiveness with profound bradycardia (30–40/min) and unmeasurable blood pressure, indicating ineffective circulation; chest compressions and endotracheal intubation begun immediately | Bolus 1: 1 mg | 1 mg |
| 12:52 | +2 min | Ventricular fibrillation identified; immediate 200-J biphasic defibrillation; CPR continued (formal cardiac arrest) | Bolus 2: 1 mg | 2 mg |
| 12:55 | +5 min | Ongoing CPR | Bolus 3: 1 mg | 3 mg |
| 12:58 | +8 min | Ongoing CPR | Bolus 4: 1 mg | 4 mg |
| 13:01 | +11 min | Ongoing CPR | Bolus 5: 1 mg | 5 mg |
| 13:04 | +14 min | Ongoing CPR | Bolus 6: 1 mg | 6 mg |
| 13:07 | +17 min | Ongoing CPR | Bolus 7: 1 mg | 7 mg |
| 13:10 | +20 min | Ongoing CPR | Bolus 8: 1 mg | 8 mg |
| 13:13 | +23 min | Ongoing CPR | Bolus 9: 1 mg | 9 mg |
| 13:16 | +26 min | Ongoing CPR | Bolus 10: 1 mg | 10 mg |
| 13:19 | +29 min | Ongoing CPR | Bolus 11: 1 mg | 11 mg |
| 13:22 | +32 min | Ongoing CPR | Bolus 12: 1 mg | 12 mg |
| 13:25 | +35 min | Ongoing CPR | Bolus 13: 1 mg | 13 mg |
| 13:28 | +38 min | Ongoing CPR; VA-ECMO cannulation completed | Bolus 14: 1 mg | 14 mg |
| 13:29 | +39 min | Full VA-ECMO flow established (37 min after confirmed arrest) | — | 14 mg |
| 13:33 | +43 min | Spontaneous sinus rhythm restored | — | 14 mg |

*Note. The no-flow interval was essentially zero: chest compressions began immediately at 12:50 upon recognition of ineffective circulation, 2 minutes before ventricular fibrillation (formal cardiac arrest) was identified at 12:52. The low-flow interval, from the onset of ineffective circulation (12:50) to full VA-ECMO flow (13:29), was 39 minutes; the interval from confirmed arrest to full VA-ECMO flow was 37 minutes. Maintenance boluses (boluses 3–14) were administered at approximately 3-minute intervals. CPR, cardiopulmonary resuscitation; VA-ECMO, veno-arterial extracorporeal membrane oxygenation.*
