# Supplementary material for: Case Report: Pulmonary reperfusion injury with bilateral bronchial cast thrombosis after VA-ECMO-bridged catheter-based reperfusion for cardiac arrest caused by high-risk pulmonary embolism
Source: Front Cardiovasc Med. 2026 Oct 5;13:1970263. doi: 10.3389/fcvm.2026.1970263 (PMC13677917; doi:10.3389/fcvm.2026.1970263)
Supplement: Supplementary file 3 [file Table3.docx]

**Supplementary Material**

*Pulmonary reperfusion injury with bilateral bronchial cast thrombosis after VA-ECMO-bridged catheter-based reperfusion for cardiac arrest caused by high-risk pulmonary embolism*

Guanshuai Chen, Fanxiang Chen, Ruoyu Li (corresponding author) — Frontiers in Cardiovascular Medicine

**Supplementary Table S3.** Serial bedside electrical impedance tomography (EIT) measurements during and after VA-ECMO support.

| **Hospital day (time)** | **ECMO status** | **Clinical context** | **Shunt%** | **DeadSpace%** | **VQMatch%** |
| --- | --- | --- | --- | --- | --- |
| Day 7 (13:50) | On VA-ECMO | Day of first catheter intervention (ECMO day 2) | 4.95 | 43.03 | 52.01 |
| Day 10 (17:26) | On VA-ECMO | Day of interval CTPA reassessment | 10.08 | 28.01 | 61.90 |
| Day 10 (20:49) | On VA-ECMO | Same-day repeat | 9.01 | 36.34 | 54.65 |
| Day 13 (00:53) | On VA-ECMO | After second intervention and first bronchoscopic clearance (day 11) | 26.22 | 1.05 | 72.73 |
| Day 13 (04:24) | On VA-ECMO | No valid measurement recorded | — | — | — |
| Day 16 (19:02) | On VA-ECMO | After second bronchoscopic cryoextraction (day 15); before weaning (day 18) | 29.32 | 11.23 | 59.45 |
| Day 19 (00:52) | Off ECMO (weaned day 18) | After third cryoextraction (day 17); first day off ECMO | 5.30 | 26.52 | 68.18 |

*Note. Hospital days are numbered from the day of admission; clock times are shown in parentheses. Shunt%, global intrapulmonary shunt fraction; DeadSpace%, dead-space ventilation fraction; VQMatch%, ventilation–perfusion match fraction; all indices were derived from hypertonic saline-bolus EIT. Because saline-bolus perfusion maps under VA-ECMO reflect residual native transpulmonary flow rather than total pulmonary perfusion, on-ECMO indices are semi-quantitative and should be interpreted as within-patient trends. —, no valid measurement recorded. CTPA, computed tomography pulmonary angiography; VA-ECMO, veno-arterial extracorporeal membrane oxygenation.*
