# Supplementary material for: Case Report: Pulmonary reperfusion injury with bilateral bronchial cast thrombosis after VA-ECMO-bridged catheter-based reperfusion for cardiac arrest caused by high-risk pulmonary embolism
Source: Front Cardiovasc Med. 2026 Oct 5;13:1970263. doi: 10.3389/fcvm.2026.1970263 (PMC13677917; doi:10.3389/fcvm.2026.1970263)
Supplement: Supplementary file 4 [file Table4.docx]

# **CARE Checklist (2013) — Completed**

**Manuscript title:** Case Report: Pulmonary reperfusion injury with bilateral bronchial cast thrombosis after VA-ECMO-bridged catheter-based reperfusion for cardiac arrest caused by high-risk pulmonary embolism

**Target journal:** Frontiers in Cardiovascular Medicine

**Corresponding author: Ruoyu Li (dgszyyzzyxkcgs@vip.163.com)**

**Reference:** Gagnier JJ, Kienle G, Altman DG, Moher D, Sox H, Riley D; the CARE Group. The CARE Guidelines: Consensus-based Clinical Case Reporting Guideline Development. J Med Case Rep (2013) 7:223. doi: 10.1186/1752-1947-7-223

## **Title — Item 1**

| Item | Checklist item description | Location / Statement |
| --- | --- | --- |
| 1 | The diagnosis or intervention of primary focus followed by the words “case report” | ☑ Title page (Page 1). Title carries the “Case Report:” prefix and states the primary focus (VA-ECMO-bridged reperfusion; pulmonary reperfusion injury with bronchial cast thrombosis) |

## **Key Words — Item 2**

| Item | Checklist item description | Location / Statement |
| --- | --- | --- |
| 2 | 2 to 5 key words that identify diagnoses or interventions in this case report (including “case report”) | ☑ Page 1: high-risk pulmonary embolism; cardiac arrest; VA-ECMO; bronchial cast thrombus; cryoextraction; electrical impedance tomography; case report |

## **Abstract — Item 3**

| Item | Checklist item description | Location / Statement |
| --- | --- | --- |
| 3a | Introduction — What is unique about this case and what does it add to the scientific literature? | ☑ Abstract, sentences 1–4 (Page 1): bronchial cast thrombosis after pulmonary reperfusion injury is rare and challenging; VA-ECMO bridging with staged catheter reperfusion described |
| 3b | The patient’s main concerns and important clinical findings | ☑ Abstract (Page 1): preoperative cardiac arrest from high-risk PE in a femoral neck fracture patient; reperfusion injury with bilateral bronchial cast thrombi; EIT monitoring |
| 3c | The primary diagnoses, interventions, and outcomes | ☑ Abstract (Page 1): VA-ECMO bridging, staged catheter thrombolysis/thrombectomy, repeated bronchoscopic cryoextraction; weaned from ECMO and ventilation; persistent vegetative state |
| 3d | Conclusion — What are one or more “take-away” lessons? | ☑ Abstract, final sentences (Page 1): vigilance for reperfusion injury and airway cast thrombosis; cryoextraction effective; EIT useful; hypoxic-ischemic brain injury may determine long-term prognosis |

## **Introduction — Item 4**

| Item | Checklist item description | Location / Statement |
| --- | --- | --- |
| 4 | Briefly summarizes why this case is unique and may include medical literature references | ☑ Section 1 (Pages 1–2), references [1]–[11]; uniqueness stated in final paragraph |

## **Patient Information — Item 5**

| Item | Checklist item description | Location / Statement |
| --- | --- | --- |
| 5a | De-identified patient specific information | ☑ Section 2.1 (Page 2): no name, ID, address, or facial data; presented only as “a woman in her 60s” (sex and approximate age band as the minimum clinically relevant information); no other identifying details |
| 5b | Primary concerns and symptoms of the patient | ☑ Section 2.1 (Page 2): left hip pain and limited mobility for 5 hours after a left-sided fall while walking; left femoral neck fracture on CT |
| 5c | Medical, family, and psychosocial history including relevant genetic information | ☑ Section 2.1 (Page 2): 6-year hypertension on telmisartan, reportedly well-controlled; no diabetes mellitus, coronary artery disease, cerebrovascular disease, or prior thromboembolic events; never-smoker, no alcohol; retired and living with her family; no family history of thromboembolic or cardiovascular disease; screening for inherited thrombophilia negative; allergies: penicillin and sulfonamides; Caprini score 10 (extremely high VTE risk) |
| 5d | Relevant past interventions and their outcomes | ☑ Section 2.1 (Page 2): long-term telmisartan with reportedly adequate control; no prior surgery; preoperative enoxaparin thromboprophylaxis; femoral neck fracture surgery pending at the time of arrest |

## **Clinical Findings — Item 6**

| Item | Checklist item description | Location / Statement |
| --- | --- | --- |
| 6 | Significant physical examination and important clinical findings | ☑ Sections 2.1–2.4 (Pages 2–3): admission cardiopulmonary and abdominal examination unremarkable; focal left-hip signs (mild swelling, maximal inguinal midpoint tenderness, palpable bony crepitus, positive heel-tap test); sudden cardiac/respiratory arrest on day 6; ECG complete right bundle branch block; bedside echocardiography showing an abnormal echogenic mass at the left pulmonary artery ostium; bloody secretions from the endotracheal tube after reperfusion; ABG deterioration |

## **Timeline — Item 7**

| Item | Checklist item description | Location / Statement |
| --- | --- | --- |
| 7 | Historical and current information from this episode of care organized as a timeline (figure or table) | ☑ Table 1: chronological clinical course, admission day 1 to day 75 (17 timepoints) |

## **Diagnostic Assessment — Item 8**

| Item | Checklist item description | Location / Statement |
| --- | --- | --- |
| 8a | Diagnostic methods (PE, laboratory testing, imaging, surveys) | ☑ Sections 2.2–2.5: ECG, bedside echocardiography, pulmonary artery CTA, DSA pulmonary angiography, ABG analysis, bedside bronchoscopy, bedside EEG, EIT (Shunt/DeadSpace/VQMatch) |
| 8b | Diagnostic challenges | ☑ Section 2.2: diagnosis established under cardiac arrest; echocardiography first suggested PE; confirmation deferred until CTA on day 7 under VA-ECMO support |
| 8c | Diagnosis (including other diagnoses considered) | ☑ Sections 2.2–2.3: echocardiography first suggested acute PE (abnormal echogenic mass at the left pulmonary artery ostium); high-risk PE confirmed by pulmonary artery CTA (bilateral main and branch pulmonary artery embolism) |
| 8d | Prognostic characteristics when applicable | ☑ Section 2.7: repeated bedside EEG with poor amplitude; day-75 EEG slow background — poor neurological prognostic signs; persistent vegetative state |

## **Therapeutic Intervention — Item 9**

| Item | Checklist item description | Location / Statement |
| --- | --- | --- |
| 9a | Types of therapeutic intervention (pharmacologic, surgical, preventive) | ☑ Sections 2.2–2.6: VA-ECMO; catheter-directed thrombolysis + aspiration/mechanical thrombectomy + balloon angioplasty; bronchoscopic forceps extraction, lavage, cryoextraction; anticoagulation; tracheostomy; hyperbaric oxygen |
| 9b | Administration of therapeutic intervention (dosage, strength, duration) | ☑ Epinephrine 1 mg ×14 boluses (first bolus 12:50 during the peri-arrest phase; second bolus 12:52 after 200-J defibrillation for ventricular fibrillation; twelve further 1-mg boluses at approximately 3-minute intervals, 12:55–13:28; cumulative 14 mg — Supplementary Table S2); defibrillation 200 J ×1; catheter-directed urokinase 200,000 U (twice, days 7 and 11); 6 mm × 40 mm balloon angioplasty; intravenous unfractionated heparin days 6–15 (targets APTT 50–70 s and bedside ACT 140–160 s, with further deliberate reduction during active bleeding); enoxaparin 2,000 U SC daily from day 16; VA-ECMO approximately 12 days |
| 9c | Changes in therapeutic interventions with explanations | ☑ Sections 2.4 and 2.6: heparin promptly reduced after the day-11 hemorrhage (APTT 39.6 s on day 12); during re-titration, APTT transiently overshot to 108 s on day 14 — recognized the same day, the heparin infusion was reduced, and APTT returned to 54.5 s the following day without recurrent bleeding (Supplementary Table S1); anticoagulation transitioned to subcutaneous enoxaparin 2,000 U daily on day 16 once airway bleeding was bronchoscopically controlled (bleeding–thrombosis balance, per ELSO guidance); remaining bronchial thrombi deferred on day 15 because further removal carried a high risk of recurrent airway bleeding |

## **Follow-up and Outcomes — Item 10**

| Item | Checklist item description | Location / Statement |
| --- | --- | --- |
| 10a | Clinician- and patient-assessed outcomes if available | ☑ Sections 2.5–2.7 and Table 1: VA-ECMO weaned day 18; tracheostomy day 28 and ventilator weaning from day 29 (Table 1); patient-assessed outcomes n/a (persistent vegetative state — family perspective reported in Section 4) |
| 10b | Important follow-up diagnostic and other test results | ☑ ABG before/after the first bronchoscopic clearance (pH 7.150→7.340; PaO₂ 52→100 mmHg; pre-procedure values in Section 2.4, post-procedure values in Table 1); EIT VQMatch% 52.01%→68.18% (day 7 vs day 19, Section 2.5 and Supplementary Table S3); day-38 brain MRI (cerebral atrophy, Fazekas grade 1) and day-75 EEG slow background (Section 2.7) |
| 10c | Intervention adherence and tolerability (how assessed) | ☑ Anticoagulation dynamically adjusted per coagulation/ACT; airway clearance completed in staged sessions due to bleeding risk (Section 2.4) |
| 10d | Adverse and unanticipated events | ☑ Pulmonary reperfusion injury with severe pulmonary hemorrhage; bilateral bronchial cast thrombosis; hypoxic-ischemic encephalopathy with persistent vegetative state |

## **Discussion — Item 11**

| Item | Checklist item description | Location / Statement |
| --- | --- | --- |
| 11a | Strengths and limitations in your approach to this case | ☑ Section 3.6 (Strengths and limitations) |
| 11b | Discussion of the relevant medical literature | ☑ Sections 3.1–3.6, references [1]–[26] (VA-ECMO bridging, structured algorithms, ELSO guidance, reperfusion injury, balloon dilation, cryoextraction, EIT, E-CPR neuroprognostication) |
| 11c | The rationale for your conclusions | ☑ Sections 3.1–3.6 and Section 5 |
| 11d | The primary “take-away” lessons (without references) in a one paragraph conclusion | ☑ Section 5 (Conclusion): bridging value of VA-ECMO; vigilance for reperfusion injury and airway cast thrombi; cryoextraction as rescue technique; EIT as bedside adjunct; neurological prognosis caveat |

## **Patient Perspective — Item 12**

| Item | Checklist item description | Location / Statement |
| --- | --- | --- |
| 12 | The patient should share their perspective on the treatment(s) received | ☑ Section 4: patient in persistent vegetative state and unable to provide a personal perspective; perspective provided by family/legal representative |

## **Informed Consent — Item 13**

| Item | Checklist item description | Location / Statement |
| --- | --- | --- |
| 13 | Did the patient give informed consent? Please provide if requested | ☑ Yes — written informed consent for publication obtained from the patient’s legal representative/next of kin (Ethics statement); signed consent form available upon request |

**Completed by:** Ruoyu Li (corresponding author), on behalf of all authors

**Date: 2026-09-24**
